# Compressed sensing MRI via fast linearized preconditioned alternating direction method of multipliers

**DOI:** 10.1186/s12938-017-0343-x

**Published:** 2017-04-27

**Authors:** Shanshan Chen, Hongwei Du, Linna Wu, Jiaquan Jin, Bensheng Qiu

**Affiliations:** 1 Center for Biomedical Engineering, Department of Electronic Science and Technology, University of Science and Technology of China, Heifei, 230027 China; 2 Department of Radiology, University of Washington School of Medicine, Seattle, WA 98108 USA; 3 Anhui Computer Application Institute of Traditional Chinese Medicine, Hefei, 230038 China

**Keywords:** Compressed sensing MRI, Image reconstruction, Total variation, Alternating direction method of multipliers

## Abstract

**Background:**

The challenge of reconstructing a sparse medical magnetic resonance image based on compressed sensing from undersampled *k*-space data has been investigated within recent years. As total variation (TV) performs well in preserving edge, one type of approach considers TV-regularization as a sparse structure to solve a convex optimization problem. Nevertheless, this convex optimization problem is both nonlinear and nonsmooth, and thus difficult to handle, especially for a large-scale problem. Therefore, it is essential to develop efficient algorithms to solve a very broad class of TV-regularized problems.

**Methods:**

In this paper, we propose an efficient algorithm referred to as the fast linearized preconditioned alternating direction method of multipliers (FLPADMM), to solve an augmented TV-regularized model that adds a quadratic term to enforce image smoothness. Because of the separable structure of this model, FLPADMM decomposes the convex problem into two subproblems. Each subproblem can be alternatively minimized by augmented Lagrangian function. Furthermore, a linearized strategy and multistep weighted scheme can be easily combined for more effective image recovery.

**Results:**

The method of the present study showed improved accuracy and efficiency, in comparison to other methods. Furthermore, the experiments conducted on in vivo data showed that our algorithm achieved a higher signal-to-noise ratio (SNR), lower relative error (Rel.Err), and better structural similarity (SSIM) index in comparison to other state-of-the-art algorithms.

**Conclusions:**

Extensive experiments demonstrate that the proposed algorithm exhibits superior performance in accuracy and efficiency than conventional compressed sensing MRI algorithms.

## Background

Magnetic resonance imaging (MRI), which is non-invasive, provides non-electromagnetic radiation, higher soft-tissue contrast, and spatial resolution, has been applied in diagnostic medicine for many years. However, due to the limitations of the hardware scanning system and the traditional Nyquist sampling theory, MRI scanners take a considerable length of time to acquire *k*-space data. Patient motion (e.g. a beating heart and respiratory movement) during lengthy scans can cause motion and streaking artifacts on the reconstructed image. This degrades image quality, which could lead to misdiagnosis. Thus, accelerating the sampling speed and reducing or eliminating artifacts have always been the aims of many studies.

With the rapid development of the novel compressed sensing (CS) theory [1, 2], compressed sensing MRI (CS-MRI) has attracted much attention, as it can reduce imaging time considerably. Compressed sensing theory claims that by using random projection, a small number of data points can be directly sampled at a sampling frequency that is far below the Nyquist frequency. In CS-MRI, the imaging time can be significantly reduced by reconstructing an image of good quality from highly undersampled *k*-space data. Specifically, if the signal or image is sparse in a certain domain, we can obtain perfect reconstruction with sufficient measurements. MR images are generally sparse in some transform domain, such as the wavelet domain. Consequently, the CS technique can be easily combined with MRI. The original signals or images can be recovered by using the nonlinear reconstruction algorithm under the restricted isometry property (RIP) [3, 4]. Nevertheless, owing to the limitations of MR physics, MRI cannot achieve two-dimentional random sampling.

In 2007, Lustig et al. [5] proposed the SparseMRI algorithm, which selects wavelet transform as a sparse basis, and uses variable density random sampling and the conjugate gradient descent method for image recovery. This was the first application of CS to MRI. However, due to a high time complexity, the SparseMRI is too slow to be put into practical use. Since then, a variety of nonlinear algorithms have been proposed for CS-MRI reconstruction. The *alternating* *direction* *method* *of* *multipliers* (ADMM) algorithm has been studied extensively [6–8], and has been widely used in optimization problems that arise in machine learning, image processing, etc. Recently, the fast alternating direction method of multipliers (FADMM) [9] has incorporated a predictor-corrector acceleration scheme into the simple ADMM, when a strongly convex condition is satisfied. This algorithm cannot guarantee a global convergence when weakly convex problems are encountered. Another fast method, referred to as the accelerated alternating direction method of multipliers (ALPADMM) [10], was proposed to deal with a class of affine equality constrained composite optimization problems. Although ALPADMM is capable of handling saddle point problems, its convergence rate largely depends on the Lipschitz constant of the smooth component.

In this article, we propose an efficient algorithm to solve an augmented total variation (TV)-regularized model that adds a quadratic term to the classical TV-regularized model to enforce smoothness of the image. The proposed method applies a linearization strategy to two quadratic terms and divides the original convex problem into two subproblems, both of which are easy to solve. For all subproblems, the augmented Lagrangian function, which combines both the Lagrangian function and the quadratic penalty function, is applied to update each variable and gain more reconstruction accuracy than the Lagrangian function approach alone. Furthermore, we utilize a multistep weighted technique to improve the accuracy of reconstruction. Numerical experiments have been conducted to compare the proposed algorithm with previous algorithms on various MR images. The experimental results show that the proposed approach can achieve a higher signal-to-noise ratio (SNR), lower relative error (Rel.Err), and better structural similarity (SSIM) index. The main contributions of the work are twofold as follows: (i) the proposed linearized preconditioned alternating direction method of multipliers (FLPADMM) that is inspired by the smooth technique [9], linearized strategy [10, 11], and the accelerated method [10] is designed to solve the augmented TV-regularized model; and (ii) this algorithm only linearizes the closed convex function and does not require the application of multistep weighting to each variable.

The paper is organized as follows: In "Related work", the CS-MRI reconstruction algorithms are reviewed. "Methods" briefly describes the basics of CS problem formulation and the proposed FLPADMM method to reconstruct MR images. The experimental results of the proposed approach and comparison with other algorithms are illustrated in "Results". Corresponding discussions are given in "Discussion" and conclusions are provided in "Conclusions".

## Related work

In this section, we briefly review the conventional CS-MRI reconstructed algorithms. Many nonlinear algorithms, e.g., the iterative shrinkage/thresholding method (IST) [12], two-step IST (TwIST) scheme [13], fast IST algorithm (FISTA) [14], split augmented Lagrangian shrinkage algorithm (SALSA) [6], wavelet tree sparsity MRI (WaTMRI) [15], total variation augmented Lagrangian alternating direction method (TVAL3) [16], total variation based compressed magnetic resonance imaging (TVCMRI) [17], reconstruction from partial Fourier data (RecPF) [18], and fast composite splitting algorithm (FCSA) [19], have been proposed to improve reconstruction speed and accuracy. IST is an operator-splitting algorithm that can be applied to an optimization problem with a simpler regularization term. The global acceleration of IST may be very slow especially when the stepsize is quite small or the optimization problem is extremely ill-conditioned. TwIST, which is a variant of IST, utilizes two or more previous iterates to update the current values, and does not depend on the previous iterate alone. TwIST gains higher speed than IST on reconstruction problem; however, the global convergence rate of TwIST has not been thoroughly studied. FISTA is another accelerated variant of IST that also takes advantage of two previous iterates. Unlike TwIST, FISTA can achieve global convergence with a splitting scheme.

SALSA transforms a constrained problem into an unconstrained problem by adding a penalty term that can be dealt with, using an augmented Lagrangian approach. Many image linear inverse problems, including image deblurring, MRI image reconstruction, and image inpainting, can be handled with SALSA. According to structured sparsity theories, WaTMRI exploits the tree sparsity to improve CS-MRI, which combines standard wavelet sparsity with total variation. To our knowledge, TVAL3 was proposed to solve a set of equality-constrained nonsmooth problems, and integrated an alternative minimization technique with a nonmonotone line search to optimize an augmented Lagrange function. Both TVCMRI and RecPF utilize a splitting strategy to minimize objective function. The former uses operator-splitting technology, and the latter adopts a variable splitting method to obtain optimal solution. In FCSA, the original optimization problem is divided into two easier subproblems that can be easily solved by FISTA. However, these algorithms are not necessarily easily implemented, or even sufficient to solve CS problems with TV-regularization, especially when the measurement matrix is considerably ill-conditioned. Therefore, it is necessary to develop algorithms that are both accurate and efficient to solve large-scale problems.

## Methods

### Problem formulation

Generally, the classical TV-regularized model for CS-MRI reconstruction problems can be written as:

1 $$\begin{aligned} \min _{x} \quad \text {TV}(x)\triangleq |\nabla x| \quad s.t. \quad \Vert Ax-b\Vert _{2} \le \delta , \end{aligned}$$

where $x \in R^{n}$ is the image to be reconstructed, $b \in R^{m}$ denotes the undersampled *k*-space data from the MR scanner, and $A\in R^{m \times n} (m < n)$ is the measurement matrix. The expression $\delta > 0$ represents the noise level in the measurement data and $(\nabla x)_{i,j} = (x_{i+1,j}-x_{i,j},x_{i,j+1}-x_{i,j})$, where $\nabla$ is the discrete gradient operator and $|\nabla x|$ denotes the TV regularization of *x*. A variant of (1) is the following TV-regularization problem:

2 $$\begin{aligned} \min _{x} \frac{1}{2} \Vert Ax-b\Vert ^2_{2}+\tau |\nabla x|, \end{aligned}$$

where $\tau > 0$ is a positive regularization parameter to balance between the two objectives. To enforce image smoothness, we add a quadratic term $\frac{\gamma }{2}\Vert \nabla x\Vert ^2_{2}$ in the objective function to give the new TV-regularized model:

3 $$\begin{aligned} \min _{x} \frac{1}{2} \Vert Ax-b\Vert ^2_{2}+\tau |\nabla x|+ \frac{\gamma }{2}\Vert \nabla x\Vert ^2_{2}, \end{aligned}$$

where $\gamma$ is a smoothing parameter. The augmented term $|\nabla x| + \frac{\gamma }{2}\Vert \nabla x\Vert ^2_{2}$ can yield accurate solutions by using a proper value of $\gamma$. In addition, the dual problem is continuously differentiable and facilitates effective use of gradient information. To split the variable *x*, an auxiliary variable *z* is introduced by $\nabla x - z = 0$, and the unconstrained optimization problem (3) is transformed into:

4 $$\begin{aligned} \min _{x} \frac{1}{2} \Vert Ax-b\Vert ^2_{2}+ \tau |z| + \frac{\gamma }{2}\Vert z\Vert ^2_{2} ,\quad s.t. \quad \nabla x - z = 0. \end{aligned}$$

The augmented Lagrangian function for problem (4) is given by:

5 $$\begin{aligned} \mathcal {L} (x,z,\uplambda) = \frac{1}{2} \Vert Ax-b\Vert ^2_{2}+ \tau |z|+\frac{\gamma }{2}\Vert z\Vert ^2_{2} - \langle\uplambda , \nabla x- z\rangle + \frac{\mu }{2}\Vert \nabla x- z\Vert ^2_{2}, \end{aligned}$$

where $\uplambda \in R^{m}$ is the Lagrangian multiplier, $\mu$ is a positive penalty parameter, and $\langle\uplambda , \nabla x- z\rangle$ denotes the inner product of the vectors $\uplambda$ and $\nabla x-z$. The classical ADMM minimizes the convex optimization problem (5) with respect to *x* and *z*, using the non-linear block–Gauss–Seidel technique. After minimizing *x* and *z* alternatively, $\uplambda$ can be updated by:

6 $$\begin{aligned} \uplambda _{k+1}=\uplambda _{k} - \mu (\nabla x_{k+1}-z_{k+1}). \end{aligned}$$

The non-linear block–Gauss–Seidel iteration of ADMM can be written as:

7 $$\begin{aligned} \left\{ \begin{array}{ll} x_{k+1} &{}\leftarrow \text {arg}\min \limits _{x}\mathcal {L} (x,z_{k},\uplambda _{k}),\\ z_{k+1} &{}\leftarrow \text {arg}\min \limits _{z}\mathcal {L} (x_{k+1},z,\uplambda _{k}),\\ \uplambda _{k+1} &{}\leftarrow \uplambda _{k} - \mu (\nabla x_{k+1}-z_{k+1}). \end{array} \right. \end{aligned}$$

Suppose $z_{k}$ and $\uplambda _{k}$ are given, then $x_{k+1}$ can be obtained by:

8 $$\begin{aligned} x_{k+1}=\text {arg}\min \limits _{x} \frac{1}{2} \Vert Ax-b\Vert ^2_{2} - \langle\uplambda _{k}, \nabla x- z_{k}\rangle + \frac{\mu }{2}\Vert \nabla x- z_{k}\Vert ^2_{2}. \end{aligned}$$

When $x_{k+1}$ and $\uplambda _{k}$ remain fixed, $z_{k+1}$ can be minimized by:

9 $$\begin{aligned} z_{k+1}=\text {arg}\min \limits _{z} \tau |z|+\frac{\gamma }{2}\Vert z\Vert ^2_{2} - \langle\uplambda _{k}, \nabla x_{k+1} - z\rangle + \frac{\mu }{2}\Vert \nabla x_{k+1} - z \Vert ^2_{2}. \end{aligned}$$

The ADMM algorithm above, used to solve (4), is expressed in Algorithm 1.
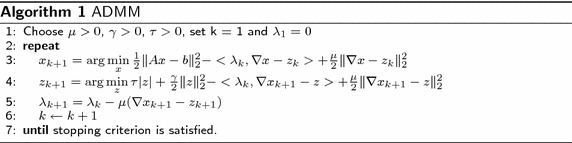


The ADMM has been previously studied and analyzed [7, 8, 20]. Generally, if subproblems in (7) are not in closed-form, many solutions could exist within those subproblems. Moreover, when the objective functions are poor or difficult to handle at high precision, the conventional ADMM algorithms might also perform poorly in image reconstruction.

### Proposed algorithm

Based on the above analysis, firstly, the minimization of (8) is given by:

10 $$\begin{aligned} x_{k + 1}&= \text {arg}\min _{x} \frac{1}{2} \Vert Ax-b\Vert ^2_{2} - \langle\uplambda _{k}, \nabla x - z_{k}\rangle + \frac{\mu }{2}\Vert \nabla x - z_{k}\Vert ^2_{2},\nonumber \\&= \text {arg}\min _{x} \frac{1}{2} \Vert Ax-b\Vert ^2_{2} + \frac{\mu }{2} \Vert \nabla x - \left(z_{k}+\frac{\uplambda _{k}}{\mu }\right)\Vert ^2_{2}. \end{aligned}$$

Hence, (10) is transformed into:

11 $$\begin{aligned} ( A^{T}A + \mu \nabla ^{T} \nabla )x_{k+1} = A^{T} b + \nabla ^{T}(\mu z_{k}+ \uplambda _{k}). \end{aligned}$$

Because the measurement matrix *A* is neither the identity matrix, nor is it typically fully dense, it is impossible to derive the exact solution with respect to the *x* vectors [21]. In addition, the computational cost to handle (11) is extremely heavy. In order to reduce the computational burden and get closed-form solutions, some variants could be considered. The linearization of the quadratic term $\frac{1}{2} \Vert Ax-b\Vert ^2_{2}$ is used to update $x_{k+1}$ as follows:

12 $$\begin{aligned} \frac{1}{2} \Vert Ax-b\Vert ^2_{2} \approx \frac{1}{2} \Vert Ax_{k}-b\Vert ^2_{2} + \langle Grad(x_{k}), x - x_{k}\rangle + \frac{\eta }{2}\Vert x - x_{k}\Vert ^2_{2}, \end{aligned}$$

where $Grad(x_{k}) = A^T(Ax_{k} -b)$ is the gradient of $\frac{1}{2} \Vert Ax-b\Vert ^2_{2}$ at the current point $x_{k}$, and $\eta$ is a positive proximal parameter. Then, the *x* subproblem in (10) can be iterated by:

13 $$\begin{aligned} x_{k + 1}&= \text {arg}\min _{x}\langle Grad(x_{k}), x - x_{k}\rangle + \frac{\eta }{2}\Vert x - x_{k}\Vert ^2_{2}\nonumber \\&- \langle \uplambda _{k}, \nabla x - z_{k} \rangle + \frac{\mu }{2}\Vert \nabla x - z_{k}\Vert ^2_{2}. \end{aligned}$$

Considering the quadratic term $\frac{\mu }{2}\Vert \nabla x - z_{k}\Vert ^2_{2}$ can also be linearized, we also linearize $\frac{\mu }{2}\Vert \nabla x - z_{k}\Vert ^2_{2}$ at $x_{k}$. This variant is a fast linearized preconditioned ADMM(FLPADMM) algorithm that generates the iterates $x_{k+1}$ by:

14 $$\begin{aligned} x_{k + 1}&= \text {arg}\min _{x} \mathcal {L} (x,z_{k},\uplambda _{k}),\nonumber \\&= \text {arg}\min _{x} \frac{1}{2} \Vert Ax-b\Vert ^2_{2} -\langle \uplambda _{k}, \nabla x - z_{k}\rangle + \frac{\mu }{2}\Vert \nabla x - z_{k}\Vert ^2_{2},\nonumber \\&= \text {arg}\min _{x} \langle Grad(x_{k}), x - x_{k}\rangle + \frac{\eta }{2}\Vert x - x_{k}\Vert ^2_{2} - \langle \uplambda _{k}, \nabla x \rangle \nonumber \\& \quad +\langle \mu (\nabla x_{k} - z_{k}), \nabla x \rangle,\nonumber \\&= \text {arg}\min _{x}\langle Grad(x_{k}), x - x_{k} \rangle + \frac{\eta }{2}\Vert x - x_{k}\Vert ^2_{2} \nonumber \\ &\quad + \langle \mu (\nabla x_{k}- z_{k}) - \uplambda _{k},\nabla x \rangle. \end{aligned}$$

The negative divergence operator $-div$ can be used to solve (14) as follows:

15 $$\begin{aligned} x_{k+1} = x_{k} - \frac{1}{\eta }(-div(\mu (\nabla x_{k} - z_{k})-\uplambda _{k})+ Grad(x_{k})). \end{aligned}$$

Secondly, for a given $x_{k + 1}$ and $\uplambda _{k}$, $z_{k+1}$ is computed by solving:

16 $$\begin{aligned} z_{k+1}&= \text {arg}\min _{z} \mathcal {L} (x_{k + 1},z,\uplambda _{k}), \nonumber \\&= \text {arg}\min _{z} \tau |z|+\frac{\gamma }{2}\Vert z\Vert ^2_{2} - \langle \uplambda _{k}, \nabla x_{k+1} - z \rangle + \frac{\mu }{2}\Vert \nabla x_{k+1} - z \Vert ^2_{2},\nonumber \\&= \text {arg}\min _{z} \tau |z| +\frac{\gamma }{2}\Vert z\Vert ^2_{2} + \frac{\mu }{2}\Vert z - \left(\nabla x_{k+1} - \frac{\uplambda _{k}}{\mu }\right)\Vert ^2_{2},\nonumber \\&= \text {arg}\min _{z} \tau |z| + \frac{\gamma + \mu }{2}\Vert z - \frac{\mu }{\gamma + \mu }\left(\nabla x_{k+1} -\frac{\uplambda _{k}}{\mu } \right)\Vert ^2_{2},\nonumber \\&= \text {arg}\min _{z} \frac{\tau }{\gamma + \mu }|z| + \frac{1}{2}\Vert z - \frac{\mu }{\gamma + \mu }\left(\nabla x_{k+1}-\frac{\uplambda _{k}}{\mu }\right)\Vert ^2_{2}. \end{aligned}$$

Hence, the solution to (16) obeys:

17 $$\begin{aligned} z_{k+1} = soft\left( \frac{\mu }{\gamma + \mu }\left( \nabla x_{k+1} - \frac{\uplambda _{k}}{\mu }\right),\frac{\tau }{\gamma + \mu }\right), \end{aligned}$$

where $soft( \cdot , T)$ is the soft thresholding function that is defined as:

18 $$\begin{aligned} soft(v, T) = \left\{ \begin{array}{ll} v + T, \quad& v < -T,\\ 0, \quad& |v| \le |T|,\\ v - T, \quad& v > T. \end{array} \right. \end{aligned}$$

Finally, the Lagrangian multiplier is updated by $\uplambda _{k+1}=\uplambda _{k} - \mu (\nabla x_{k+1}-z_{k+1})$.

The FLPADMM algorithm utilizes the gradient descent method and one soft thresholding operator to update variables at each iteration. In addition, this method is a variant of the classical ADMM algorithm framework. The proposed FLPADMM algorithm is presented in Algorithm 2.
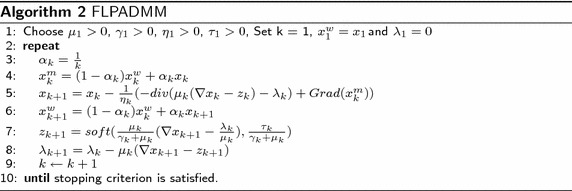


The stopping criterion in all of the algorithms above is the relative change of *x* between two successive iterations, which is small enough, i.e.:

19 $$\begin{aligned} \frac{\Vert x_{k+1}-x_{k}\Vert _{2}}{\Vert x_{k}\Vert _{2}} \le tol \end{aligned}$$

where *tol* is usually a range chosen from $10^{-5}$ to $10^{-3}$. In the FLPADMM algorithm, $\alpha _{k}$ represents a weighted parameter and $Grad(x^{m}_{k})$ is the gradient of $\frac{1}{2} \Vert Ax-b\Vert ^2_{2}$ at the point $x^{m}_{k}$. Furthermore, $x^{m}_{k}$ is the first weighted value that is used to update $x_{k+1}$.

It should be noted that the appropriate parameter $\alpha _{k}$ can improve the rate of convergence that has been proven in a previous study [10]. Moreover, the superscript *m* stands for “middle,” and *w* stands for “weight.” Before the gradient descent method in line 5 is applied, *x* is updated by the weighted sums of all previous iterates. Furthermore, after the gradient method is applied, *x* is updated again by the same weighting technique, that is, *x* is weighted twice at each iteration. Specifically, when the weighted parameter $\alpha _{k}$ is set to 1, the *x* subproblem is simply the current point $x_{k+1}$. At this point, FLPADMM becomes another variant of the ADMM algorithm [10]. The accelerated strategy of FLPADMM incorporates a multistep acceleration scheme with middle point $x^{m}$ and weighted point $x^{w}$, which was first applied in a previous study [10] and derived from the accelerated gradient method [22, 23]. Moreover, the optimal rate of convergence of FLPADMM is $O\left(\frac{1}{k^{2}} + \frac{1}{k}\right)$.

## Results

### Experimental setup

**Fig. 1:**
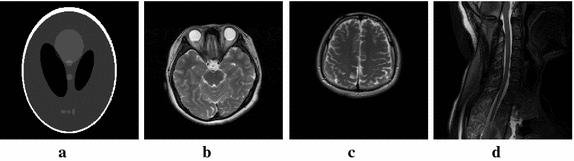
MR images. (**a**) Shepp–Logan phantom (**b**) human brain1 image (**c**) human brain2 image (**d**) human spine image

A series of numerical experiments were conducted to compare the performance of the proposed FLPADMM with two state-of-the-art algorithms, namely FADMM [9] and ALPADMM [10], for MR image reconstruction from undersampled *k*-space data. The four typical MR datasets (Shepp–Logan phantom, human brain1 MR data, human brain2 MR data, and human spine MR data) were used to evaluate our algorithm. All test images had the same matrix size of 256 × 256, as shown in Fig. 1a–d. The first Shepp–Logan phantom was a piecewise smooth image with pixel intensities ranging from 0 to 1. The complex *k*-space data of the human brain1 was acquired from a 3T GE MR750 scanner using the FRFSE sequence (TR = 6000 ms, TE = 101 ms). The human brain2 data was also obtained from the 3T GE MR750 system (TR/TE = 2500/96.9 ms, field of view = 280 × 280 mm, slice thickness = 5 mm). The human spine MR data was fully sampled *k*-space data acquired on a 3T GE MR750 system with a FRFSE sequence (TR/TE = 2500/110 ms, field of view = 240 × 240 mm, slice thickness = 5 mm). To achieve fair comparisons, codes of all compared algorithms were downloaded from the authors’ websites. All experiments were executed, using Windows 10 and MATLAB 2015b (64-bit), on a desktop computer with a 3.2GHz Intel core i5-4460 CPU and 8GB of RAM.

**Fig. 2:**
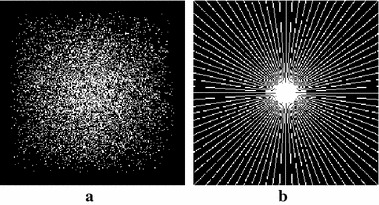
Sampling masks.** a** pseudo-Gaussian mask at 15% sampling rate,** b** pseudo-radial mask at 18% sampling rate

Each experiment was repeated 10 times, and the average image metric results of 10 experiments were recorded. For most of the MR images, the *k*-space signals with a large magnitude were generally localized in the central region. Since a non-Cartesian sampling matrix is incoherent, the results on the Cartesian masks were far less favorable than those on the non-Cartesian mask. Therefore, two non-Cartesian masks were chosen as the sampling masks. One was a pseudo-Gaussian mask, displayed in Fig. 2a, that was implemented by following the sampling strategy of collecting more low-frequency signals in the central part of the *k*-space, and less high-frequency signals in the peripheral part of the *k*-space. The other mask presented in Fig. 2b, was a pseudo-radial mask that was applied by following the rule of RecPF [18]. The sampling ratio was defined as the number of sampled points divided by the total size of the original image.

**Fig. 3:**
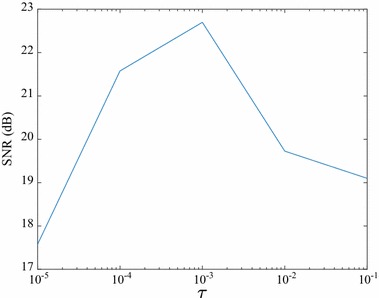
Analysis to determine the optimum regularization parameters for human brain1 data using a pseudo-Gaussian mask at a sampling rate of 25%

In the present study, we compared our algorithm with two state-of-the-art algorithms under similar conditions. To explore the influence of the regularization parameter $\tau$, we used human brain1 data as an example, to analyze the changes of image quality when $\tau$ was changed. In Fig. 3, the SNR attained the maximum value when $\tau$ was 10^−3^. Thus, we chose this optimum value to achieve favorable reconstruction. Similar searches were adopted for the other datasets. For all tests, we also found that when $\gamma = 2\tau$, our algorithm maintained the most favorable reconstruction performance. Furthermore, the default maximum of all three methods was set to 300.

To quantitatively evaluate the result of the proposed algorithm, three objective metrics were adopted to measure the quality of the recovered images. The first was SNR, defined as:

20 $$\begin{aligned} \text {SNR}=10\log _{10}\frac{\sum _{i=0}^{M}\sum _{j=0}^{N}x(i,j)^2}{\sum _{i=0}^{M}\sum _{j=0}^{N}(x(i,j)-\overset{\wedge }{x}(i,j))^2}, \end{aligned}$$

where *x* is the original image, $\overset{\wedge }{x}$ is the reconstructed image, and *M* and *N* represent the number of rows and columns, respectively, in the input image. The quality of the reconstructed image is directly proportional to the value of SNR. The second metric was the Rel.Err, defined as:

21 $$\begin{aligned} \text {Rel.Err}=\frac{\Vert x-\overset{\wedge }{x}\Vert _{2}}{\Vert x\Vert _{2}}\times 100\%. \end{aligned}$$

A smaller value meant that the reconstructed image had little error and more favorable reconstruction in comparison to the original image.

The last metric was the SSIM index that was used to measure the similarity between two images, in terms of structure, brightness, and contrast, among other aspects, and defined as:

22 $$\begin{aligned} \text {SSIM(p,q)}=\frac{(2\mu _{p}\mu _{q}+c_{1})(2\theta _{pq}+c_{2})}{(\mu ^2_{p}+\mu ^2_{p}+c_{1})(\theta ^2_{p}+\theta ^2_{q}+c_{2})}, \end{aligned}$$

where $\mu _{p}$ and $\theta _{p}$ are the mean and variance, respectively, of the original image; $\mu _{q}$ and $\theta _{q}$ are the mean and variance, respectively, of the reconstructed image, $\theta _{pq}$ is the covariance of these two images; and $c_{1}$ and $c_{2}$ are fixed constants that prevent unstable phenomena when the denominator is close to zero. When the value of SSIM was increased, the image showed greater similarity to the original.

### Experimental results

**Fig. 4:**
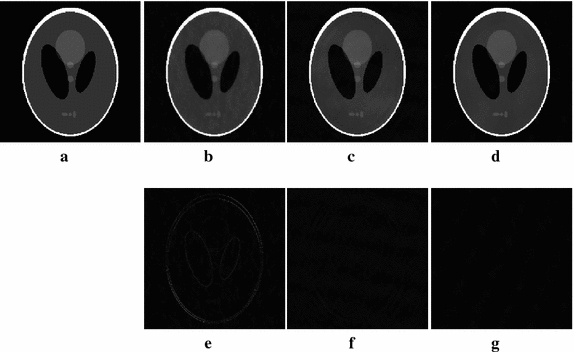
Results of three methods under a pseudo-Gaussian mask with 20% sampling.** a** Original Shepp–Logan phantom,** b** FADMM,** c** ALPADMM,** d** proposed FLPADMM, and** e**–**g** *error map* of** b**–**d**, respectively

In this section, we first compare our proposed FLPADMM algorithm with FADMM [9] and ALPADMM [10] algorithms on the Shepp–Logan phantom, with Gaussian white noise of a standard deviation of 0.01. The proposed FLPADMM was applied to the Shepp–Logan phantom under pseudo-Gaussian mask with 20% *k*-space data undersampled. Figure 4a shows the original Shepp–Logan phantom, and Fig. 4b–d presents the reconstructed images recovered by the FADMM, ALPADMM, and FLPADMM algorithms, respectively. Compared with the original Shepp–Logan phantom image, FADMM yielded noticeable artifacts and failed to suppress background noise. The image recovered by ALPADMM contained fewer artifacts and was evidently more favorable than that recovered by FADMM. As the Shepp–Logan phantom is extremely piecewise smooth and sparse, ALPADMM also provides good reconstruction. As shown in Fig. 4c, d, visible artifacts are not easily observed when both ALPADMM and FLPADMM are used. However, for experiments on in vivo data as we will show, FLPADMM would perform much more accurately and stably than ALPADMM.

For enhanced visualization, Fig. 4e–g depicts the difference between the reconstructed image and the original image of the Shepp–Logan phantom under a pseudo-Gaussian mask at a sampling ratio of 20% using FADMM, ALPADMM, and the proposed FLPADMM reconstruction. It was evident that the reconstruction with FLPADMM had the smallest error. The proposed FLPADMM exhibited superior performance in suppressing noise without significant artifacts, and yielded the best reconstruction.

**Fig. 5:**
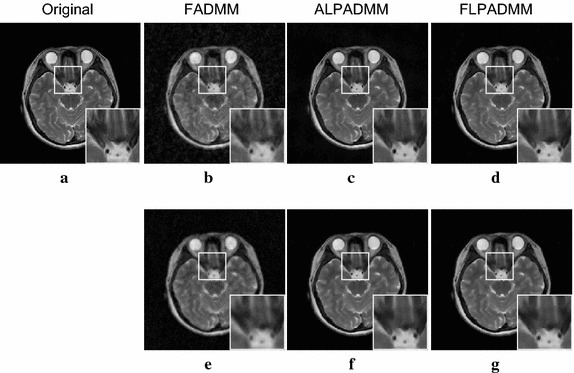
Reconstructed images and zoomed-in regions among the state-of-the-art MR image reconstruction algorithms using a pseudo-Gaussian mask (*first row*) and pseudo-radial mask (*second row*) with 25% sampling.** a** Original human brain1 image,** b**,** e** FADMM,** c**,** f** ALPADMM,** d**,** g** FLPADMM

**Fig. 6:**
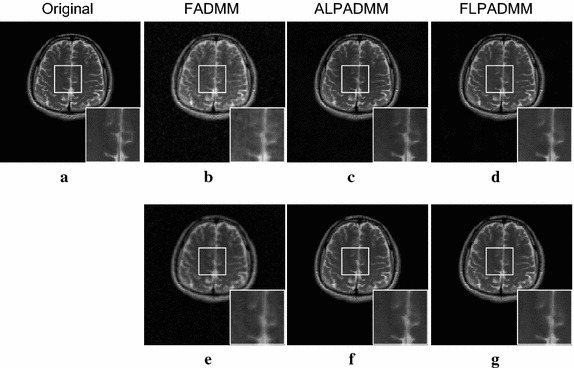
Reconstructed images and zoomed-in regions among the state-of-the-art MR image reconstruction algorithms using a pseudo-Gaussian mask (*first row*) and pseudo-radial mask (*second row*) with 25% sampling.** a** Original human brain1 image,** b**,** e** FADMM,** c**,** f** ALPADMM,** d**,** g** FLPADMM

All experiments on in vivo data were corrupted with Gaussian white noise with zero mean and a standard deviation of 0.01. Experimental results of these in vivo human brain data are displayed in Figs. 5 and 6 at a sampling ratio of 25%. Figure 5a presents the original human brain1 image. The reconstructed images illustrated in Fig. 5b–d, were obtained by FADMM, ALPADMM, and our proposed FLPADMM, under a pseudo-Gaussian sampling scheme. Figure 5e–g was produced by FADMM, ALPADMM, and FLPADMM, respectively, under a pseudo-radial sampling pattern. The SNR of the human brain1 image under the pseudo-Gaussian mask using FLPADMM was 25.0685 dB, whereas those recovered by FADMM and ALPADMM were 20.9921 and 22.3231 dB, respectively. We can clearly see that the FLPADMM reconstruction suppressed background noise. The recovery result in Fig. 6 is similar to that in Fig. 5.

**Fig. 7:**
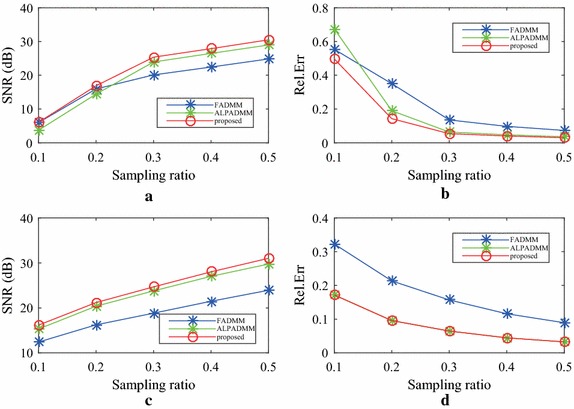
Comparison results of human brain1 data using a pseudo-Gaussian mask (*first row*) and pseudo-radial mask (*second row*).** a**,** c** SNR (dB) vs sampling ratio,** b**,** d** Rel.Err vs sampling ratio

Figure 7 gives the comparison results of human brain1 data among the state-of-the-art MR image reconstruction algorithms, using different sampling masks, when the sampling ratios were 0.1, 0.2, 0.3, 0.4, and 0.5, respectively. As seen in Fig. 7a, b, the proposed FLPADMM achieved high image quality with high SNR and low Rel.Err. When the sampling ratio was 0.1, the three methods performed relatively poorly. That is because when sampling ratio is too low, the sampled data is insufficient to obtain a faithful image. It is notable that as the sampling ratio increased for all algorithms under consideration, the SNR was also increased, whereas Rel.Err was gradually reduced. That is, the reconstructions of higher quality could have been obtained by taking more measurements. In addition, when the sampling ratio was increased, the FLPADMM algorithm exhibited superior performance in recovering the sampling image. Specifically, a sampling ratio of 30% was sufficient to reconstruct the human brain1 image effectively.

**Fig. 8:**
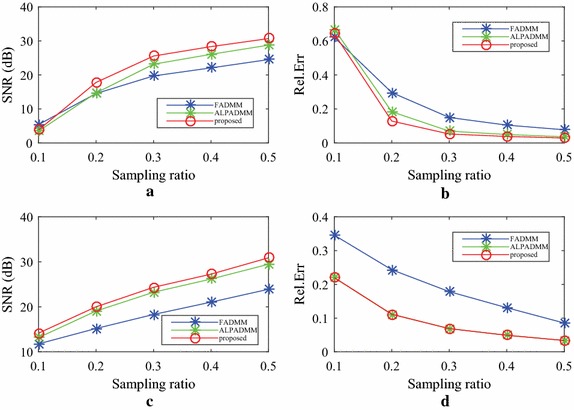
Comparison results of human brain2 data using a pseudo-Gaussian mask (*first row*) and pseudo-radial mask (*second row*).** a**,** c** SNR (dB) vs sampling ratio,** b**,** d** Rel.Err vs sampling ratio

**Fig. 9:**
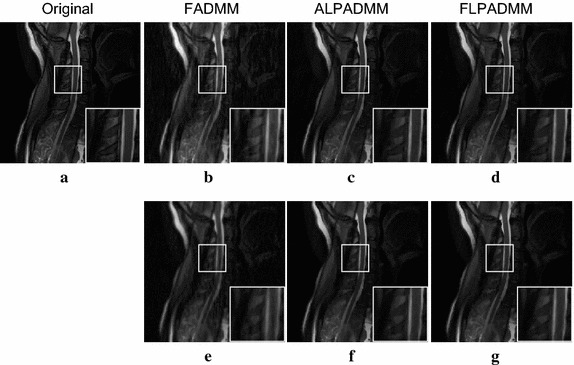
Reconstructed images and zoomed-in regions among the state-of-the-art MR image reconstruction algorithms using a pseudo-Gaussian mask (*first row*) and pseudo-radial mask (*second row*) with 25% sampling.** a** Original human brain1 image,** b**,** e** FADMM,** c**,** f** ALPADMM,** d**,** g** FLPADMM

**Fig. 10:**
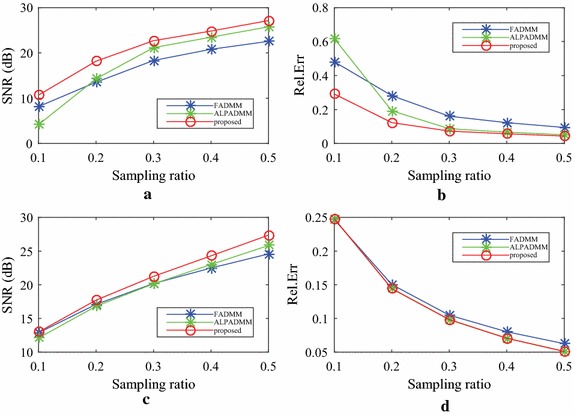
Comparison results of human spine data using a pseudo-Gaussian mask (*first row*) and pseudo-radial mask (*second row*).** a**,** c** SNR (dB) vs sampling ratio,** b**,** d** Rel.Err vs sampling ratio

Figure 8 shows the comparison results of human brain2 data, where (a) and (c) present the SNR of the human brain2 image with different ratios, and (b) and (d) describe the Rel.Err of the human brain2 image at different ratios. The results are similar to those of the human brain1 image. The SNR of FLPADMM was slightly larger than that of ALPADMM when a pseudo-radial mask was applied. The Rel.Err of ALPADMM was very close to that of FLPADMM (see Figs. 7d, 8d), indicating that both ALPADMM and our proposed FLPADMM show similar recovery performance under the pseudo-radial mask.

**Table 1:** Additional reconstruction results on different MR images with a pseudo-Gaussian mask under different sample ratios

Test image	Metric	Algorithm	Sampling ratio				
			0.1	0.2	0.3	0.4	0.5
Human brain1	SSIM	FADMM	0.3493	0.4497	0.7052	0.9707	0.9842
		ALPADMM	0.1470	0.5126	0.9748	0.9836	0.9904
		FLPADMM	0.3981	0.5241	0.9788	0.9869	0.9918
	CPU time (s)	FADMM	2.9609	3.0238	3.0955	3.1921	3.6528
		ALPADMM	4.2605	4.3840	4.4864	4.6043	4.7250
		FLPADMM	4.0840	4.3312	4.4342	4.5427	4.5791
Human brain2	SSIM	FADMM	0.2786	0.3702	0.7391	0.9828	0.9914
		ALPADMM	0.1631	0.4244	0.9877	0.9922	0.9955
		FLPADMM	0.3325	0.4215	0.9911	0.9944	0.9961
	CPU time (s)	FADMM	3.0527	3.2095	3.2354	3.2802	3.2332
		ALPADMM	4.3250	4.4526	4.7770	4.5932	4.6251
		FLPADMM	4.2333	4.4974	4.5447	4.4557	4.6193
Human spine	SSIM	FADMM	0.2077	0.3270	0.4755	0.5528	0.6200
		ALPADMM	0.4396	0.7779	0.9425	0.9672	0.9807
		FLPADMM	0.6235	0.7869	0.9731	0.9830	0.9889
	CPU time (s)	FADMM	2.9718	3.0915	3.1162	3.1401	3.1521
		ALPADMM	4.3979	4.4857	4.6409	4.5899	4.6300
		FLPADMM	4.3400	4.4285	4.4914	4.5481	4.6091

The CPU time and SSIM comparison among FADMM, ALPADMM, and the proposed FLPADMM

**Table 2:** Additional reconstruction results on different MR images with a pseudo-radial mask under different sample ratios

Test image	Metric	Algorithm	Sampling ratio				
			0.1	0.2	0.3	0.4	0.5
Human brain1	SSIM	FADMM	0.4324	0.5741	0.7739	0.7794	0.8468
		ALPADMM	0.7023	0.8810	0.9397	0.9714	0.9835
		FLPADMM	0.6647	0.9512	0.9691	0.9854	0.9919
	CPU time (s)	FADMM	2.8064	2.8258	2.9255	2.9367	3.0155
		ALPADMM	4.2838	4.3129	4.3371	4.4753	4.4069
		FLPADMM	4.2480	4.2847	4.3309	4.4369	4.3676
Human brain2	SSIM	FADMM	0.4602	0.5735	0.6748	0.7869	0.8608
		ALPADMM	0.6803	0.8928	0.9535	0.9828	0.9909
		FLPADMM	0.6460	0.9767	0.9874	0.9944	0.9972
	CPU time (s)	FADMM	2.8830	2.8922	2.9648	3.0214	3.0325
		ALPADMM	4.2863	4.5635	4.3734	4.5279	4.6064
		FLPADMM	4.2239	4.3988	4.3313	4.4163	4.5621
Human spine	SSIM	FADMM	0.6269	0.7439	0.8288	0.8885	0.9276
		ALPADMM	0.7531	0.8861	0.9373	0.9657	0.9801
		FLPADMM	0.7677	0.9325	0.9630	0.9794	0.9887
	CPU time (s)	FADMM	2.8823	2.8769	2.9891	3.2540	3.0932
		ALPADMM	4.4947	6.2550	6.4025	6.4653	6.5581
		FLPADMM	4.3113	4.4198	4.3130	4.3558	4.5122

The CPU time and SSIM comparison among FADMM, ALPADMM, and the proposed FLPADMM

The reconstructed results in Fig. 9 were consistent with those of Fig. 5. Compared to FADMM and ALPADMM, our proposed FLPADMM reconstructed better images without visual artifacts. For example, when the sampling ratio was 25% under pseudo-radial sampling, FADMM had significant artifacts and ALPADMM had slight artifacts. However, images reconstructed by FLPADMM were the closest to the original image of the human spine. These results further validate the superiority of FLPADMM in comparison to other algorithms and are consistent with the results of the two human brain experiments. It is clear that regardless of the sampling scheme, FLPADMM achieved the highest SNR and lowest Rel.Err. From Fig. 10, we can see that all reconstruction results showed steady improvement as the sampling ratio increased. Moreover, FLPADMM showed superior performance in comparison to other algorithms. As the tissue structure of the human spine MR data was extremely complex, the quality of the reconstructed image was not as good as that of the human brain tests.

For further comparison, the results of quantitative image metrics, SSIM and CPU time (s), are listed in Tables 1 and 2 to demonstrate structural similarity and running time of FADMM, ALPADMM, and FLPADMM algorithms for all MR images at various sampling ratios. As seen in Table 2, the running time of FLPADMM was faster than that of ALPADMM, but slower than that of FADMM by approximately 1 s. Since the convergence of FADMM was also close to $O(\frac{1}{k^2})$ when strict conditions were satisfied, the running time of all three methods were very similar. According to the value of the SSIM, the proposed FLPADMM achieved higher quality images than the other techniques.

## Discussion

We proposed a novel algorithm for CS-MRI reconstruction, referred to as FLPADMM. Except for the TV-regularization term in the classical MR model, we added a quadratic term to this classical model to make the image smoother. Using augmented Lagrangian function, FLPADMM effectively divides the original convex problem into two subproblems, both of which can be easily dealt with. To further enhance image reconstruction, a strategy that incorporated a multistep weighted scheme was adopted in FLPADMM. Several parameters need to be tuned in our proposed algorithm. In general, the requirement on stepsize $\eta$ obeys $\eta > \mu \Vert A^{T}A\Vert$. When the regularization parameter $\tau$ is $10^{-3}$ (see Fig. 3) and $\gamma = 2\tau$, our proposed algorithm yields the best result. The other parameters can be manually set for different test data under a fixed sampling scheme. When different sampling schemes (i.e., pseudo-Gaussian mask and pseudo-radial mask) are applied, our proposed FLPADMM can also produce very impressive results. Experiments validate that the performance of this proposed method is superior to those of FADMM and ALPADMM. It particularly shows the best performance in suppressing background noise, even at a low sampling ratio.

Some algorithms that combine parallel MRI and CS have been proposed to accelerate MRI reconstruction [24–26]. Our method can also be applied to parallel MRI with minor revisions.

## Conclusions

The consideration of TV-regularization for CS-MRI has been studied within recent years, largely because MR images can be recovered from its partial Fourier samples, and TV shows better performance in preserving image edges. In this paper, we first briefly reviewed nonlinear algorithms for CS-MRI, and then introduced an augmented TV-regularized model with an additional quadratic term to enforce image smoothness. An efficient, inexact, but unique algorithm has been proposed to handle this novel TV-regularized model. The proposed algorithm, referred to as FLPADMM, belongs to the classical ADMM framework that decomposes the objective function into two subproblems by adding new variables and constraints. FLPADMM minimizes the TV-regularized objective function by an augmented Lagrangian minimization function technique. Furthermore, this method effectively adopts a multistep weighted scheme to improve the accuracy of reconstruction. Moreover, FLPADMM could also solve both constrained and unconstrained convex optimization problems. Numerous experiments demonstrate the superiority of the proposed FLPADMM method in comparison to the previous FADMM and ALPADMM algorithms. Our future work would combine this algorithm with parallel MRI to further accelerate the imaging time.

## Abbreviations

MRI: magnetic resonance imaging
CS: compressed sensing
ADMM: alternating direction method of multipliers
TV: total variation
RIP: restricted isometry property
IST: iterative shrinkage/thresholding algorithm
TwIST: two-step iterative shrinkage/thresholding algorithm
FISTA: fast iterative shrinkage/thresholding algorithm
SALSA: split augmented Lagrangian shrinkage algorithm
WaTMRI: wavelet tree sparsity MRI
TVAL3: total variation augmented Lagrangian alternating direction algorithm
RecPF: reconstruction from partial Fourier data
TVCMRI: total variation based compressed MRI
FCSA: fast composite splitting algorithm
FADMM: fast alternating direction method of multipliers
ALPADMM: accelerated linearized preconditioned alternating direction method of multipliers
FLPADMM: fast linearized preconditioned alternating direction method of multipliers
SNR: signal-to-noise ratio
Rel.Err: relative error
SSIM: structural similarity index
